# Histone Deacetylase Inhibitors Selectively Target Homology Dependent DNA Repair Defective Cells and Elevate Non-Homologous Endjoining Activity

**DOI:** 10.1371/journal.pone.0087203

**Published:** 2014-01-23

**Authors:** Stephanie Smith, Jennifer Fox, Marco Mejia, Wanvipa Ruangpradit, Alihossein Saberi, Sunmi Kim, Yongjun Choi, Sehyun Oh, Yucai Wang, Kyungho Choi, Lei Li, Eric A. Hendrickson, Shunichi Takeda, Mark Muller, Kyungjae Myung

**Affiliations:** 1 Genome Instability Section, Genetics and Molecular Biology Branch, National Human Genome Research Institute, National Institutes of Health, Bethesda, Maryland, United States of America; 2 Department of Molecular Biology and Microbiology, College of Medicine, University of Central Florida, Orlando, Florida, United States of America; 3 Department of Radiation Genetics Kyoto University, Medical School, Kyoto, 606-8501 Japan; 4 Department of Environmental Health School of Public Hearth, Seoul National University, Seoul, Korea; 5 Department of Experimental Radiation Oncology, The University of Texas MD Anderson Cancer Center, Houston, Texas, United States of America; 6 Department of Genetics, The University of Texas MD Anderson Cancer Center, Houston, Texas, United States of America; 7 The University of Texas Graduate School of Biomedical Sciences at Houston, Houston Texas, United States of America; 8 Department of Biochemistry, Molecular Biology and Biophysics, University of Minnesota Medical School, Minneapolis, Minnesota, United States of America; Chang Gung University, Taiwan

## Abstract

**Background:**

We have previously used the ATAD5-luciferase high-throughput screening assay to identify genotoxic compounds with potential chemotherapeutic capabilities. The successful identification of known genotoxic agents, including the histone deacetylase inhibitor (HDACi) trichostatin A (TSA), confirmed the specificity of the screen since TSA has been widely studied for its ability to cause apoptosis in cancer cells. Because many cancers have acquired mutations in DNA damage checkpoints or repair pathways, we hypothesized that these cancers may be susceptible to treatments that target compensatory pathways. Here, we used a panel of isogenic chicken DT40 B lymphocyte mutant and human cell lines to investigate the ability of TSA to define selective pathways that promote HDACi toxicity.

**Results:**

HDACi induced a DNA damage response and reduced viability in all repair deficient DT40 mutants although *ATM*-nulls were least affected. The most dramatic sensitivity was observed in mutants lacking the homology dependent repair (HDR) factor *BLM* or the non-homologous end-joining (NHEJ) and HDR factors, *KU/RAD54*, suggesting an involvement of either HDR or NHEJ in HDACi-induced cell death. To extend these findings, we measured the frequencies of HDR and NHEJ after HDACi treatment and monitored viability in human cell lines comparably deficient in HDR or NHEJ. Although no difference in HDR frequency was observed between HDACi treated and untreated cells, HDR-defective human cell lines were clearly more sensitive than wild type. Unexpectedly, cells treated with HDACis showed a significantly elevated NHEJ frequency.

**Conclusions:**

HDACi targeting drugs induced significant increases in NHEJ activity in human cell lines but did not alter HDR frequency. Moreover, HDR is required for cellular resistance to HDACi therapy; therefore, NHEJ does not appear to be a critical axis for HDACi resistance. Rather, HDACi compounds induced DNA damage, most likely double strand breaks (DSBs), and HDR proficiency is correlated with cell survival.

## Introduction

DNA double strand breaks (DSBs) are toxic lesions that represent a major threat to cell survival. Homologous recombination (HR), also known as homology dependent repair (HDR) and non-homologous end joining (NHEJ) are two major pathways that repair DNA DSBs [1], [2], [3]. The HDR pathway, which is largely considered an error free process, functions mainly in the S and G2 phases of the cell cycle when a sister chromatid is available for use as a template [4]. In eukaryotes, the RAD51 strand exchange protein is a central player in HDR and mediates the process of synapse from a wild type allele {for review see [2]}. Other HDR proteins generally function as positive or negative regulators of RAD51. Positive regulators include BRCA1 and BRCA2. BRCA1 binds directly to the DSB, participates in the processing of the DSB by promoting 5′-end resection, and is required for the recruitment of RAD51 [4]. BRCA2 promotes RAD51-mediated strand exchange, thus controlling the assembly of the nucleofilament required for synapse [4]. Negative regulators of HDR such as BLM and FANCM have evolved to prevent uncontrolled or unscheduled HDR [5], which could ultimately lead to genomic DNA rearrangements [2]. BLM [2], RecQ5 [6], and FANCJ [7] helicases were reported to destabilize RAD51-coated single-stranded DNA (ssDNA) and FANCM is a translocase that disrupts intermediate RAD51-coated D-loops [2]. Recently, PARI [8] and RTEL [9] have been suggested as putative homologs to the yeast anti-recombination protein, Srs2. Given the importance of HDR in genome stability, it is not surprising that many of the proteins involved in HDR have been found to increase cancer susceptibility when mutated. Mutations in BRCA2 and/or BRCA1 increase predisposition to breast and ovarian cancers [4], while mutations in BLM or FANCM are associated with syndromes showing elevated genomic instability and cancer susceptibility in general [2], [10].

NHEJ is an error-prone pathway whereby the two broken ends of the DSB are conjoined by direct ligation without regard to sequence homology, but generally involve microhomology of a few bases. Whereas HDR is the major repair mechanism in the S and G2 phases of the cell cycle, the NHEJ pathway is primarily active in G1 in part because the repair protein 53BP1 binds to the broken ends and prevents resection, thereby repressing an early key step in HDR [3]. During NHEJ, the KU70-KU80 heterodimer (Ku) holds the broken ends in direct proximity and recruits other NHEJ proteins including the DNA-dependent protein kinase catalytic subunit (DNA-PKcs), Artemis, and DNA Ligase IV (LIGIV). DNA-PKcs, Ku and the DNA at the site of the DSB together form an active kinase complex (DNA-PK). Ku, Artemis, XRCC4 and LIGIV are all targets of DNA-PK, although phosphorylation of these proteins is, surprisingly, dispensable for NHEJ *in vivo* [11], [12], [13]. In contrast, autophosphorylation of DNA-PKcs is required for proper NHEJ [14]. Artemis and Ku participate in the processing of the DSB, and then the heterodimeric complex of XRCC4 and LIGIV ligate the broken DNA ends [15]. As with HDR, inappropriate NHEJ is detrimental to the cell and results in chromosomal deletions, insertions or translocations, especially when more than one DSB is available for repair [16]. Because Ku spatially stabilizes DSB ends, its loss can also lead to inappropriate joining outcomes [16]. Mutations in *ARTEMIS* are associated with severe combined immunodeficiency and predisposition to lymphomas [16]. Germ-line mutations in *LIGIV* result in LIG4 syndrome and predispose individuals to lymphoid malignancies [16]. Lastly, mutations of *DNA-PKcs* cause severe combined immunodeficiency in mice [17], [18], [19], [20]. In animals, NHEJ appears to be the major pathway for DSB repair while HDR, a high fidelity process, is much more limited. Recent evidence has suggested that the DNA synthesis associated with HDR can also reprogram DNA methylation signatures in the repaired segment, thereby leading to silencing of tumor suppressor genes or activation of oncogenes in daughter cells [21], [22], [23].

We recently developed a robust ATAD5-luciferase high-throughput screening (HTS) assay based on the stabilization of the DNA damage response protein ATAD5 to identify genotoxic compounds and potential chemotherapeutic agents that act by inducing DNA damage [24], [25]. The ATAD5-luciferase HTS is a tractable cell based screen that identified histone deacetylase inhibitors (HDACis) as potent DNA damaging agents [25]. There is significant general interest in epigenetic therapeutics and these agents are currently under intense investigation for potential use as anti-cancer drugs [26]. HDACi therapeutics increase histone acetylation levels by inhibiting deacetylation of histones thus modifying the chromatin structure and regulating gene expression [27]. HDACis are highly pleiotropic and have numerous non-histone targets including p53, NF-kB and Rb/E2F showing widespread effects [26]. In the case of p53, this tumor suppressor protein is stabilized and transcriptionally activated by hyperacetylation, which in turn activates DNA repair or pro-apoptotic proteins [28]. Induction of cell cycle arrest, apoptosis and differentiation, coupled with inhibition of metastasis and angiogenesis, all combine to give HDACis a diverse set of anti-cancer abilities [27], [28]. TSA has been widely studied and shown to increase apoptosis in a variety of cancer types. TSA also increases the effectiveness of platinum-based therapies in human bladder cancer cells [29]. Another HDACi suberoylanilide hydroxamic acid (SAHA), has been extensively researched and was approved by the FDA in 2006 for use in the treatment of cutaneous T cell lymphoma [28]. Currently HDACis are being chemically modified to form new compounds combining the properties of the HDACis and other anti-cancer agents [27].

Because DNA repair pathways are well conserved evolutionarily we employed a panel of isogenic chicken DT40 B lymphocyte cell lines to investigate whether HDACis specifically kill DNA repair-deficient cells. Ease of target integration, a short doubling time and an unusually long S phase are all characteristics that make DT40 cells ideal for studying DNA repair and genotoxicity [30]. Here, we further examine the role that DNA repair pathways play in HDR, a pivotal pathway in defining resistance to HDACi; however, two different HDACi drugs strongly stimulate the process of NHEJ. Thus, elevation of an error prone pathway may aid in cytotoxicity of HDACi in cancer.

## Results and Discussion

**Figure 1:**
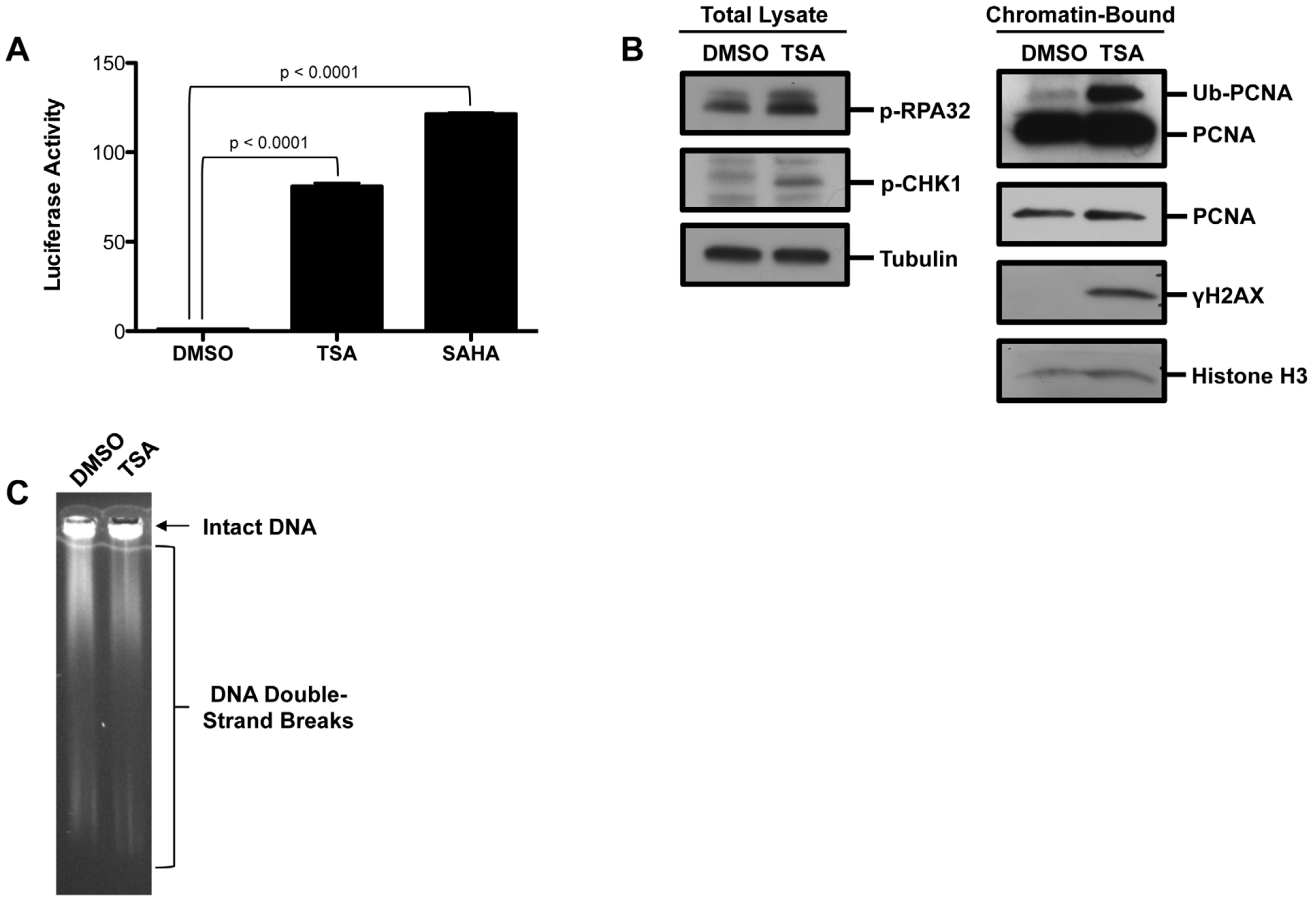
Trichostatin A induces DNA damage. (A) HEK293T ELG1-LUC cells were treated with 0.75 µM TSA or 50 µM SAHA for 48 hr. LUC activity was measured using One-Glo. The graph represents average LUC activity ± SD. Significance was calculated using a two-tailed t-test. (B) HEK293T cells were treated with 50 µM TSA for 24 hr. Using the indicated antibodies proteins levels were visualized by Western blot analysis from either the chromatin-bound fraction or total cell lysate. (C) HEK293T cells were treated with 100 uM TSA for 24 hours and then Pulsed-field gel electrophoresis was performed.

**Figure 2:**
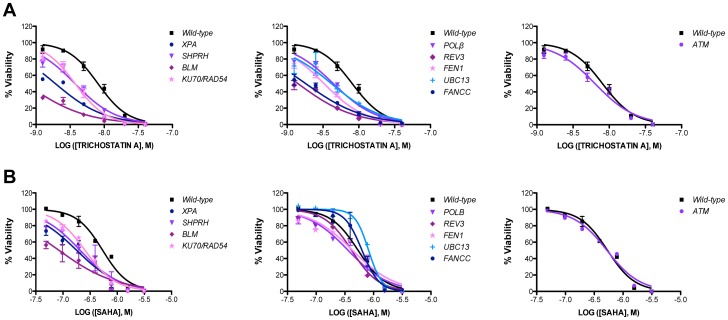
Viability of isogenic chicken DT40 B lymphocyte cell lines following 48(A) 0 to 0.04 µM TSA and (B) 0 to 3.125 µM SAHA. Viability was determined using CellTiter-Glo. The data represent the average of 3 independent experiments ± SD.

Previously we developed a high-throughput screen that used ATAD5 as a biomarker to detect genotoxic compounds, and described a preliminary screen of small chemical libraries [25]. In this screen, we found that HDACis, including TSA, increased the protein level of ATAD5 {Figure 1a and [25]} and activated DNA damage responses (Figure 1b). In addition to an increased level of γH2AX protein, pulsed-field gel electrophoresis (PFGE) confirmed a slight increase in DNA DSBs after treatment with TSA indicated by shorter DNA fragments. Many transformed cells are defective in repairing certain types of DNA damage due to deficiencies in DNA repair pathways. Transformed cells are usually highly susceptible to HDACi drugs such as TSA or SAHA [31] which also induce DNA damage (Figure 1b); thus, we reasoned that DNA repair pathways such as HDR and NHEJ are likely important in defining drug sensitivity in tumors. To test this, a panel of isogenic chicken DT40 B lymphocyte cell lines, each of which is defective in a different DNA repair pathway (Table 1), was screened for viability after treatment with increasing concentrations of TSA. The viability of the DT40 cells was assessed 48 hr after treatment, using the CellTiter-Glo luminescent cell viability assay to detect ATPase activity. Compared to wild-type, all mutant DT40 cell lines tested displayed some degree of sensitivity to TSA (Figure 2a and Table 2). *ATM*-deficient cells were not as strongly affected as other nulls but were still sensitized to TSA (Figure 2a and Table 2). To determine if the effect was specific to TSA, we also measured the viability of DT40 cell lines treated with increasing concentrations of another HDACi, SAHA (Figure 2b). The toxicity profiles of cells treated with either TSA or SAHA fell into three groups. *XPA*-, *SHPRH*-, *BLM*-, and *KU70/RAD54*-null cells fell into the first group of cells sensitive to both TSA and SAHA. *POLβ*-, *REV3*-, *FEN1*-, *UBC13*- and *FANCC*-null cells fell into the second group and only exhibited sensitivity to TSA. Finally, *ATM*-null cells were in a group by themselves and exhibited sensitivity similar to wild-type in the presence of both TSA and SAHA (Figure 2a and b and Table 2). It appeared that DNA damage caused by TSA requires all of the DNA repair pathways with the possible exception of the ATM-dependent DNA damage response, which is not thought to be required in HDR in normal mouse cells [32]. Also, base excision repair, the fanconi anemia pathway, and translesion synthesis polymerases appeared to be dispensable for the repair of SAHA-induced DNA damage.

**Table 1:** Cell lines used in this study.

Cell Type	Mutation/Name	Pathway or Description	Origin
Chicken DT40	Wild-type		Evans *et al.* 2010
Chicken DT40	*XPA*	Nucleotide Excision Repair	Evans *et al.* 2010
Chicken DT40	*SHPRH*	DNA Post-replication Repair	This study
Chicken DT40	*BLM*	DNA Replication and Repair	Evans *et al.* 2010
Chicken DT40	*FANCC*	Interstrand crosslink Repair	Evans *et al.* 2010
Chicken DT40	*KU70/RAD54*	Non-homologous End Joining/Homologous Recombination	Evans *et al.* 2010
Chicken DT40	*POLβ*	Base Excision Repair	Evans *et al.* 2010
Chicken DT40	*REV3*	Translesion DNA Synthesis	Evans *et al.* 2010
Chicken DT40	*FEN1*	Base Excision Repair, Nucleotide Excision Repair, DNA Replication	Evans *et al.* 2010
Chicken DT40	*UBC13*	DNA Post-replication Repair	Evans *et al.* 2010
Chicken DT40	*ATM*	Cell Cycle Checkpoint	Evans *et al.* 2010
Human Lymphoblast	Wild-type		Candotti *et al.* 1997
Human Lymphoblast	*BLM*	DNA Replication and Repair	Coriell
Human Lymphoblast	*XPA*	Nucleotide Excision Repair	Coriell
Mouse Embryonic Fibroblast	Wild-type		Krijger *et al.* 2011
Mouse Embryonic Fibroblast	*SHPRH*	DNA Post-replication Repair	Krijger *et al.* 2011
Human Embryonic Kidney	HEK293T		ATCC
Human Embryonic Kidney	HEK293T ELG1-LUC		Fox *et al.* 2012
Human Cervical Adenocarcinoma	HeLa		ATCC
Human Cervical Adenocarcinoma	HeLa IHN20.22	Non-homologous End Joining Assay Cell Line	This study
Human Cervical Adenocarcinoma	HeLa IHN20.41	Non-homologous End Joining Assay Cell Line	This study
Human Cervical Adenocarcinoma	HeLa iHO4.5GFP	Homologous Recombination Assay Cell Line	This study
Human Colorectal Cancer	HCT116		ATCC
Human Colorectal Cancer	HCT116 *FANCM*	Interstrand Crosslink Repair	Wang *et al.* 2013
Human Colorectal Cancer	HCT116 *LIG4*	Non-homologous End Joining	Fattah *et al.* 2010Oh *et al.* 2013
Human Ductal Carcinoma	*BRCA1* Corrected		Yu *et al.* 2006
Human Ductal Carcinoma	*BRCA1*	Homologous Recombination	Yu *et al*. 2006
Human Osteosarcoma	U2OS DR-GFP	Homologous Recombination Assay Cell Line	Xia *et al.* 2006
Human Osteosarcoma	U2OS EJ2-GFP	Alternative End Joining Assay Cell Line	Gunn *et al.* 2012
Human Retinal Pigmented Epithelium	RPE		ATCC

**Table 2:** EC50 values for isogenic chicken DT40 B lymphocyte cell lines following 48

	EC50	
	TSA nM	SAHA µM
Wild-type	8.1	0.54
*ATM*	6.4	0.53
*POLβ*	4.4	0.36
*UBC13*	4.1	0.82
*KU70/RAD54*	3.7	0.25
*SHPRH*	3.7	0.21
*FEN1*	3.2	0.82
*XPA*	1.9	0.18
*FANCC*	1.8	0.63
*REV3*	1.4	0.41
*BLM*	0.7	0.08

**Figure 3:**
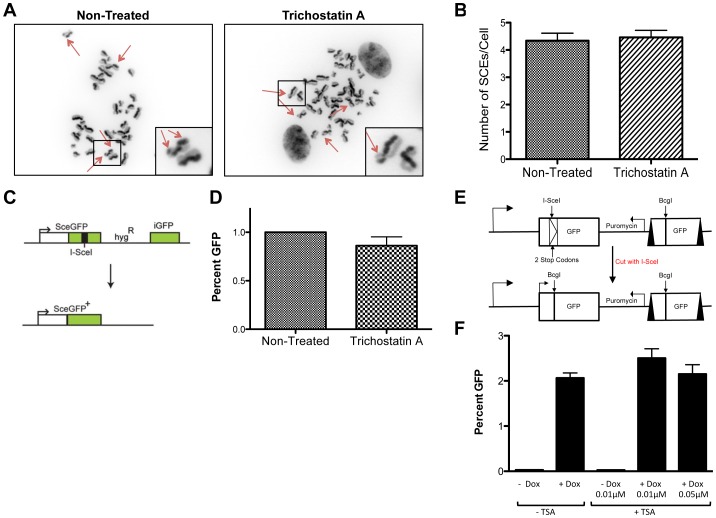
The effect of HDACis on SCE and HR Frequency. (A) RPE cells were treated with 0.2 µM TSA for 48 hr. Representative images of metaphase spreads with arrows indicating SCEs (Magnification: 100×). (Inset) Magnification of the boxed region. (B) The graph represents the average number of SCEs per cell ± SD following treatment. Non-significance was determined using a two-tailed t-test. (C) Schematic of DR-GFP HR frequency assay. (D) U2OS DR-GFP cells were transfected with pCGA-I-SCEI and then treated with 0.25 µM TSA for 48 hr. The graph represents the average HR frequency ± SD following treatment determined by FACS analysis. Non-significance was determined using a two-tailed t-test. (E) Schematic of twin reporter DR-GFP HR assay. (F) HeLa iHO4.5GFP cells were pulsed for 24 hr with Dox, washed and chased in the presence of the indicated amounts of TSA. The percentage of GFP-positive cells was determined by FACS analysis. The graph represents the average percent GFP ± SD. The difference between +Dox/−TSA and +Dox/+TSA not significant and was determined using a two-tailed t-test.

*KU70/RAD54*- and *BLM*-null DT40 cells were the most sensitive to both TSA and SAHA treatment compared to the other mutant cell lines. *KU70/RAD54*-null cells are NHEJ and HDR defective (NHEJ, due to the absence of *KU70* and HDR due to the loss of *RAD54*) [2], [3]. In contrast, *BLM*-null cells exhibit a hyper-recombination phenotype [33]. To test the possibility that HDACi treatment causes promiscuous HDR, we measured the frequency of sister chromatid exchange (SCE) in human retinal pigment epithelial (RPE) cells after TSA treatment. The frequency of SCE events for both treated and control-treated cells was not significantly different (Figures 3a and b). We also performed a single DSB-induced HDR assay using DR-GFP as a reporter in human U2OS cells [34] and found that there was no difference in HDR frequency between TSA-treated and control-treated cells (Figures 3c and d). A similar and complementary approach was performed using a HeLa cell line (iHO4.5GFP) containing twin DR-GFP reporter cassettes [35] that undergo HDR following I-SceI-induced DSB formation in the 5′ cassette [23]. In iHO4.5GFP cells, the I-SceI gene is under the control of the tet-on promoter which activates upon exposure to doxycycline (Figure 3e). Using this assay, we found that there was no significant difference in the HDR frequency of TSA or SAHA-treated cells compared to control-treated cells (Figures 3f and Figure S1a). It is possible that the decrease in HDR frequency and clear toxicity observed at higher doses of TSA (data not shown) resulted from a suppression of HDR [36], [37]. Taken together, these data indicate that treatment with TSA or SAHA does not increase the frequency of HDR. Indirect evidence that HDACi drugs do not induce promiscuous HDR repair comes from iHO4.5GFP uninduced (minus Dox) cells treated with TSA. If TSA or SAHA directly or indirectly caused genome wide DNA damage and HDR in the DR-GFP reporter, we would expect to see an increase in the percent GFP readout in the absence of I-SceI as some DNA damage and HDR should be randomly directed to the DR-GFP locus. As shown in Figure 3f and Figure S1a, there was no obvious increase in the percentage of GFP positive cells in uninduced iHO4.5GFP cells treated with HDACi alone.

**Figure 4:**
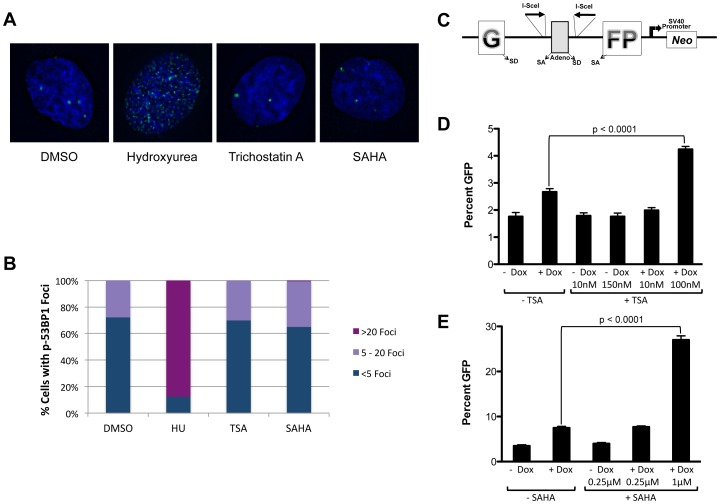
The effect of HDACi on NHEJ. (A) Representative images of RPE nuclei (blue) with p-53BP1 foci (green) after a 24 hr treatment with 2 mM HU or 1 hr treatment with either 0.5 µM TSA or 32 µM SAHA. (B) The percentage of RPE cells containing the indicated number of p-53BP1 foci following treatment as described in A was determined from at least 145 cells per treatment. (C) Schematic of NHEJ assay. HeLa IHN20.22 cells were pulsed for 24 hr with Dox, washed and chased in the presence of the indicated amounts of TSA (D) or SAHA (E) for 3 days. The graphs represent the average percent GFP ± SD. Significance between +Dox/−TSA or SAHA and +Dox/+TSA or SAHA was calculated using a two-tailed t-test. The percentage of GFP-positive cells was determined by FACS analysis.

The protein 53BP1 is recruited to DSBs and inhibits the 5′ to 3′ single-strand resection that initiates HDR [38]. ATM and DNAPKcs, which are both required for NHEJ, phosphorylate 53BP1 and p-53BP1 is a good marker for NHEJ initiation and suppression of HDR. Human RPE cells were treated with TSA, SAHA, DMSO as a negative control or hydroxyurea (HU) as a positive control and the formation of p-53BP1 foci was monitored by fluorescence microscopy. After a 1-hr treatment, there was no difference in the number of p-53BP1 foci per cell in cells treated with TSA, SAHA or DMSO (Figures 4a and b). Even after a 24-hr treatment, we did not observe any difference in p-53BP1 foci in TSA- or SAHA-treated cells compared to DMSO (data not shown). In contrast, the majority of HU-treated cells had more than 20 p-53BP1 foci (Figures 4a and b). We also used a HeLa cell-based assay to measure the frequency of NHEJ with a dedicated GFP reporter that cannot repair I-SceI DSBs by HDR (Figure 4c). IHN20.22 cells contain a single copy of a GFP cassette under the control of a CMV promoter that has been disrupted by an internal adenoviral exon. Flanking the adenoviral exon are twin I-SceI sites placed in an inverted orientation. Digestion with I-SceI results in incompatible ends that must be repaired by NHEJ [39]. These cells also have one stable copy of a cassette that encodes for doxycycline-induced I-SceI expression. Using this assay we found NHEJ to be significantly increased in cells treated with higher concentrations of TSA or SAHA compared to non-treated cells (Figures 4d and e and Figure S2a). We performed an additional NHEJ assay using the U2OS cell line EJ2-GFP [40]. Unlike the previous NHEJ assay, digestion with I-SceI in this cell line generates compatible ends that are repaired by NHEJ. Using this assay, we found that treatment with 100 nM TSA increased NHEJ frequency although not at a statistically significant level (Figure S2b). Reduction of 53BP1 by siRNA did not increase end joining of compatible ends (Figure S2c and d). Finally, we note that SAHA treated IHN20.22 cells in the absence of I-SceI cleavages (no Dox treatment) display rather large increases in NHEJ frequency as determined by increases in the percentage of GFP positive cells (Figure S2e). From this result we infer that HDACi induced DNA damage involves the generation of DSBs that are sensed and acted upon by the NHEJ pathway. Based on independent and complementary approaches, we conclude that HDACi treatment increased the frequency of NHEJ at the DSB sites; however, we did not observe enhanced 53BP1 recruitment to chromatin as noted (Figures 4a and b). The recruitment of p-53BP1 into sites of DNA damage, in particular at DSBs, involves histone H4 acetylation [41] and others have shown that HDACi treatment can suppress 53PB1 focus formation [42].

**Figure 5:**
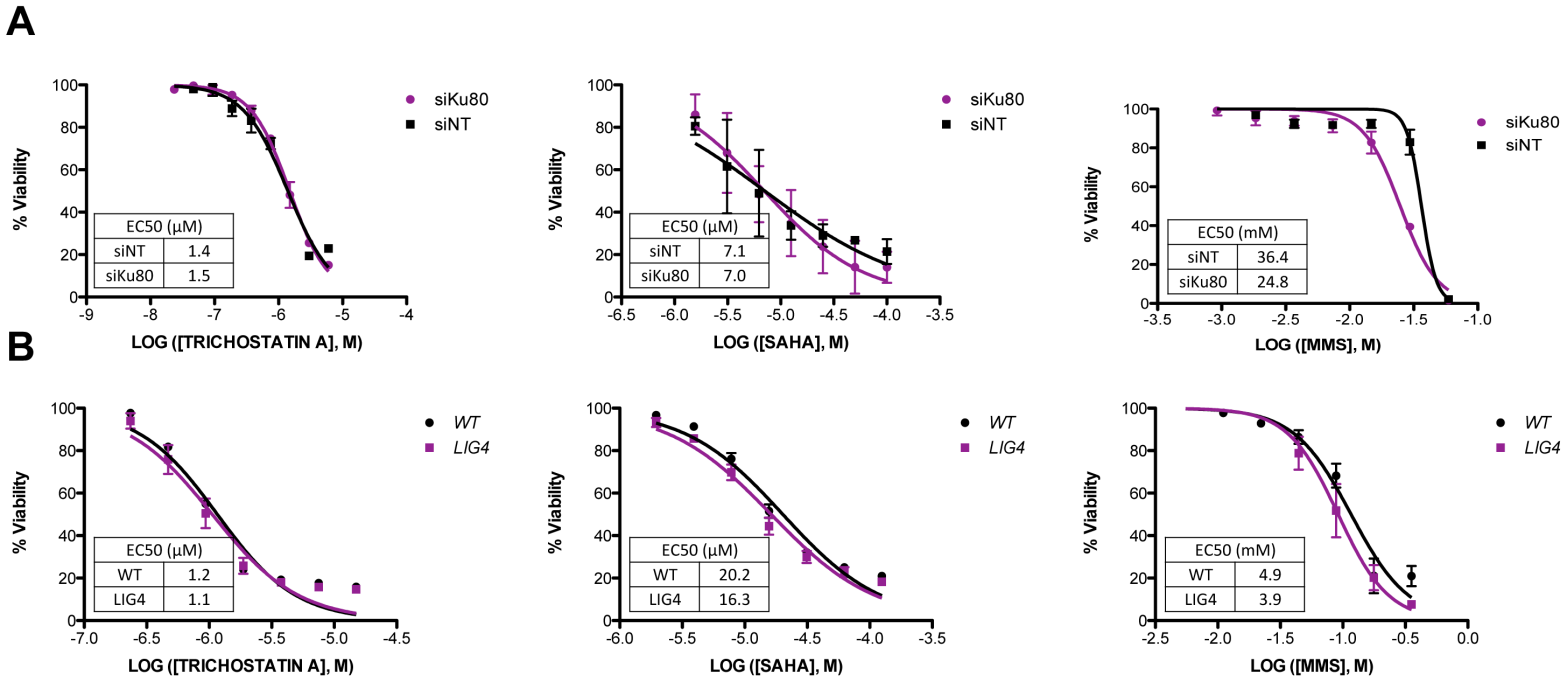
Viability of NHEJ deficient cells after treatment with HDACis. The indicated cells were treated for 48 µM TSA, 0 to 100 µM SAHA or 0 to 118 mM MMS. Viability was determined using CellTiter-Glo. The data represent the average of 3 independent experiments ± SD. (A) Hela cells after 24 hr transfection with either non-targeting siRNA or siRNA against *KU80*. (B) HCT116 *LIG4*-null or wild-type HCT116 cells. (C) HCC1937 cells, lacking a functional *BRCA1* gene, or HC1937 cells complimented by *BRCA1* cDNA expression (wild-type).

**Figure 6:**
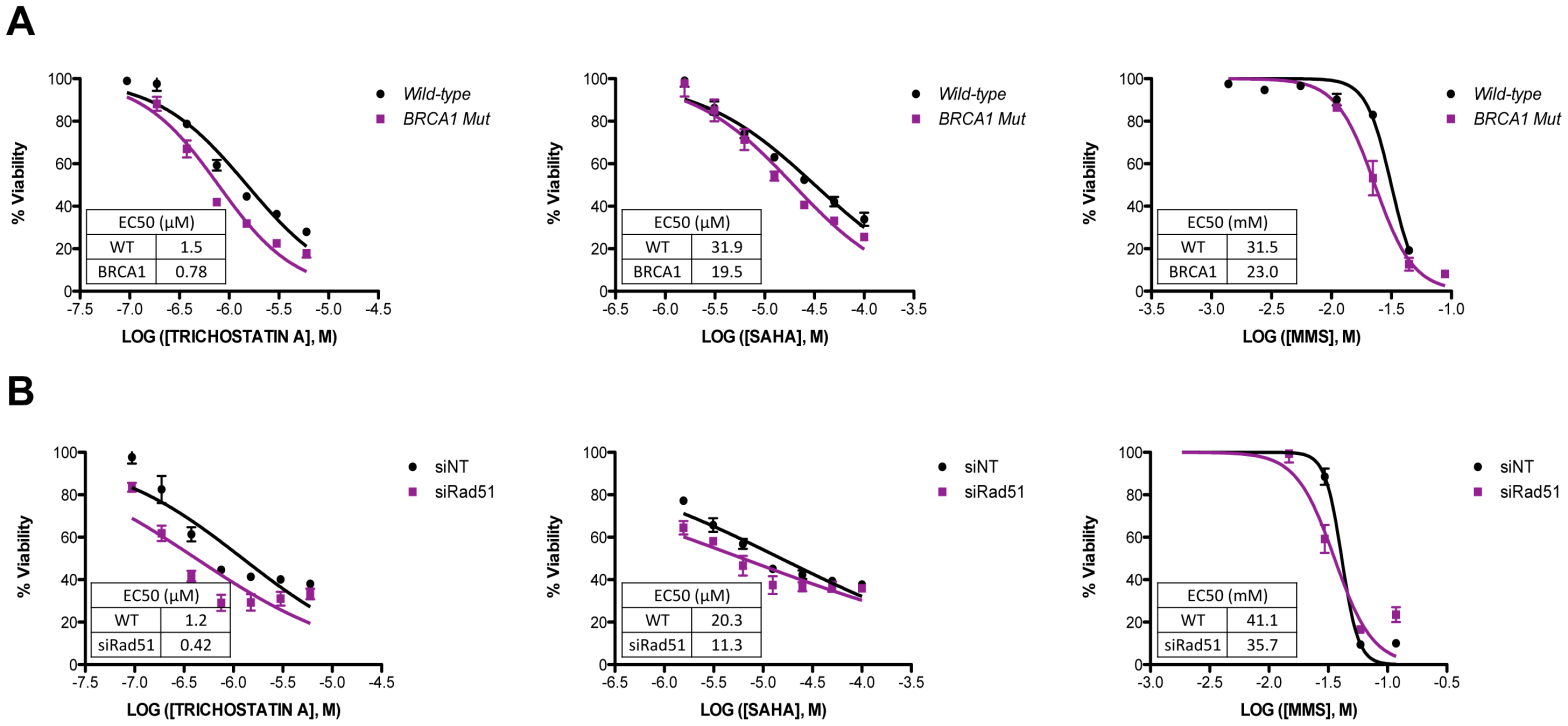
Viability of HDR deficient cells after treatment with HDACis. The indicated cells were treated for 48 µM TSA, 0 to 100 µM SAHA or 0 to 118 mM MMS. Viability was determined using CellTiter-Glo. The data represent the average of 3 independent experiments ± SD. (A) HCC1937 cells, lacking a functional *BRCA1* gene, or HC1937 cells complimented by *BRCA1* cDNA expression (wild-type). EC50 differences between wild-type and *BRCA1* after treatment with TSA and SAHA were significant at p = 0.0009 and p = 0.02 respectively. EC50 differences between wild-type and *BRCA1* after MMS treatment were not significant p = 0.07. (B) Hela cells after 2×24 hr transfection with either non-targeting siRNA or siRNA against *RAD51*. EC50 differences between siNT and siRAD51 after treatment with TSA and SAHA were significant at p<0.0001 and p = 0.03 respectively. EC50 differences between siNT and siRAD51 after MMS treatment were not significant p = 0.07.

Differences between avian and human cells could account for our inability to detect an increase in HDR frequency; therefore, we analyzed viability profiles in mammalian cell models. Logistically, this is difficult since many of the comparable human cell lines are not available. Nonetheless, to determine if human cells defective in NHEJ were sensitive to HDACi, we knocked down *KU80* expression in HeLa cells with siRNA, and then treated the cells with increasing concentrations of TSA, SAHA or, as a positive control, MMS for 48 hr. Whereas *KU86*-knockdown cells exhibited a reduced viability after treatment with MMS compared to control knockdown cells (p = 0.03 for EC50s), there was no difference in viability between control and *KU80*-knockdown cells after treatment with TSA or SAHA even though we achieved an almost complete reduction in KU80 protein level (Figure 5a and Figure S1d). In addition, we treated *LIG4*-null HCT116 cells, which are severely defective in NHEJ [43], [44], with increasing concentrations of TSA, SAHA or MMS and monitored viability. *LIG4*-null cells are sensitive to a wide range of DNA damaging agents [44] and they were therefore slightly more sensitive to MMS treatment compared to wild type although not at a statistically significant level (Figure 5b). However, we did not observe any reduction in viability of *LIG4*-null cells compared to HCT116 after treatment with either TSA or SAHA (Figure 5b). TSA can therefore enhance NHEJ frequency at DSBs, but NHEJ is dispensable for cell survival after TSA-induced DNA damage. Likewise, HCC1937 cells, which lack a functional *BRCA1* gene and are therefore defective in HDR [45], were treated with TSA, SAHA or MMS and viability was assessed after 48 hr. Compared to the control cells that were complemented by *BRCA1* cDNA expression (wild-type), *BRCA1* deficient HCC1937 cells exhibited sensitivity to TSA and SAHA that was similar to that of MMS (Figure 6a). To determine if HDR is indeed necessary to repair HDACi induced damage, we knocked down *RAD51* expression with siRNA in HeLa cells and then treated with increasing concentrations of TSA, SAHA or MMS and measured viability after 24 hours. Compared to the control knockdown cells, cells with a reduced expression of *RAD51* were more sensitive to both TSA and SAHA (Figure 6b and Figure S1e). RAD51 protein levels were only reduced by one-third, compared to control knockdown cells, which may have resulted in a decreased sensitivity to TSA and SAHA. Although we did not observe the large reduction in viability that was seen in DT40 cells, the sensitivity of *BRCA1*-deficient cells and *RAD51* deficient cells to HDACi and MMS is mutually consistent with the notion that HDR is required to repair HDACi-induced damage.

**Figure 7:**
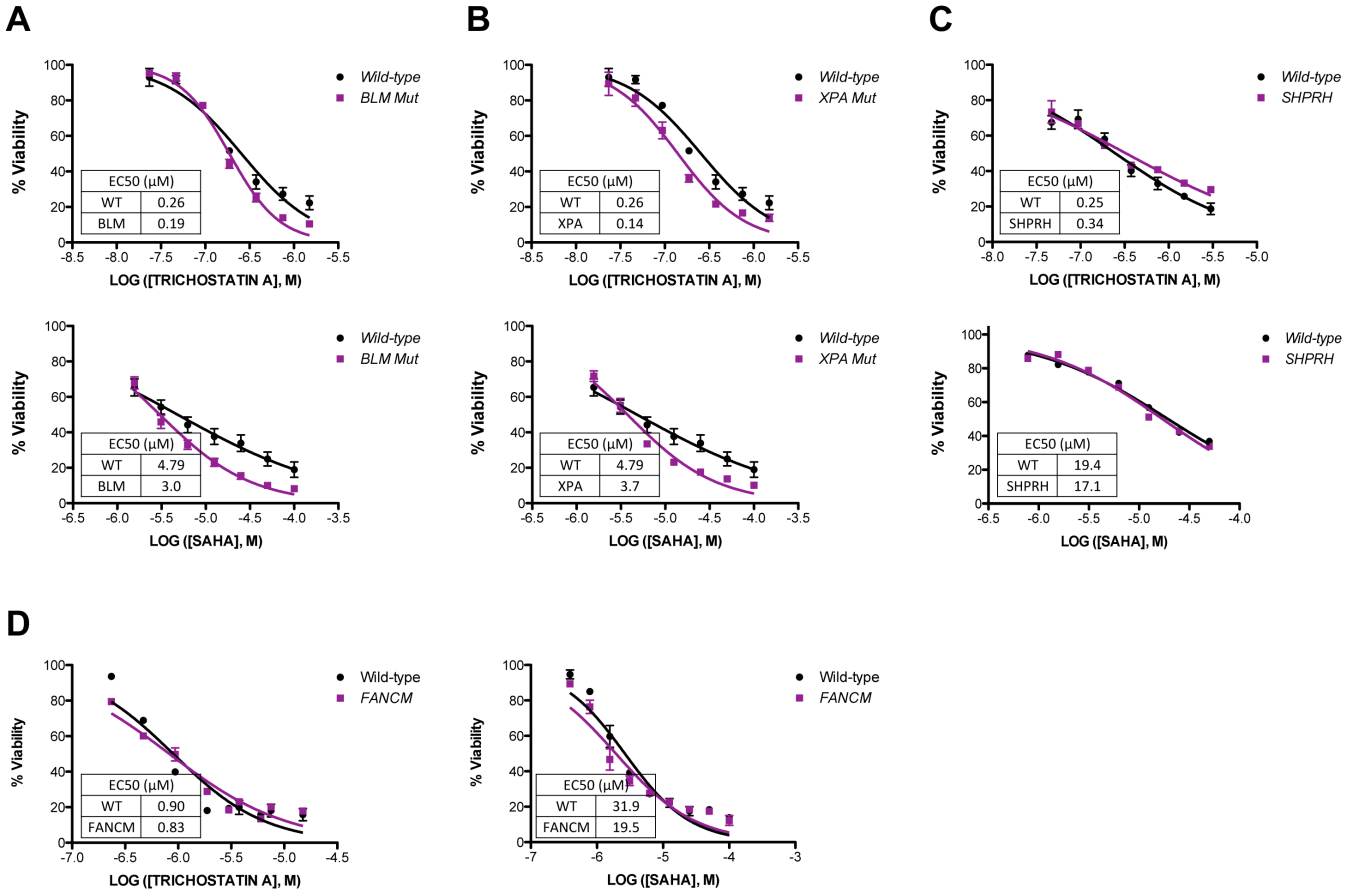
Effect of HDACi on the viability of mammalian cell lines. (A) *BLM* and (B) *XPA* human lymphocytes were treated with 0 to 1.5 µM TSA or 0 to 100 µM SAHA for 48 hours. (C) *SHPRH* MEFs were treated with 0 to 1.5 µM TSA or 0 to 100 µM SAHA for 48 hours. (D) *FANCM* human colorectal cancer cells were treated with 0 to 1.5 µM TSA or 0 to 100 µM SAHA for 48 hr. Viability was determined using CellTiter-Glo. The data represent the average of 3 independent experiments ± SD.

Because the results of our experiments with *KU70/RAD54*-null avian cells were also slightly different from what we observed using mammalian cell lines, we repeated viability assays using mammalian cell lines lacking *XPA*, *BLM*, *SHPRH*, *or FANCM* [46]. We chose to test these cell lines because the *XPA*-, *BLM*- *SHPRH*- and *FANCC*-null avian cells were some of the most sensitive to either TSA, SAHA or both. After a 48-hr treatment, *XPA*- and *BLM*- mutant human lymphocytes displayed a mild sensitivity to TSA (Figures 7a and b). EC50 values for *XPA*- and *BLM*-mutants were significantly different than wild-type (p = 0.009 and p = 0.02, respectively). Although the EC50 values for SAHA treated *XPA*- and *BLM*-mutants were not significantly different, there was a clear sensitivity of the mutants at lower survival percentages (Figures 7a and b). *XPA*- and *BLM*-mutants were both more sensitive to MMS compared to wild-type (Figure S1b). This result was similar to that observed in *BRCA1*-deficient cells (Figure 6a) with the sensitivity differences between wild-type and mutant reduced compared to what was observed in DT40 cells. *SHPRH*-null MEFs did not show any sensitivity to either TSA or SAHA when compared to wild-type MEFs (Figure 7c) and likewise, we did not observe any difference in viability between *FANCM*-deficient HCT116 cells compared to the control (Figure 7d) following treatment with TSA or SAHA. As expected *FANCM*-null cells were highly sensitive to treatment with MMS (Figure S1c). We concluded that there are slight differences in the sensitivity of avian and mammalian cells to HDACi therapeutics, but reaction to HDACi treatment also varies widely between human tumors [47] and the differences we observed might not be species specific. Collectively, however, our data also indicate that TSA and SAHA can be used to enhance the killing of cancer cells defective in specific DNA repair pathways including those dependent on BRCA1 or Rad51 (HDR), XPA (NER), or BLM.

The fact that NHEJ repair events are significantly enhanced by HDACi strategies suggests that an error prone pathway of break repair may contribute to toxicity by this class of drugs. In fact, it has been shown that high doses of TSA induce aneuploidy in lymphoblasts [48]. In marked contrast, the HDR pathway may reconcile HDACi induced genotoxicity and provide a mechanism that promotes survival of those cells that have this pathway intact. The potential for HDACi drugs to target cells with HDR defects and stimulate NHEJ events is significant. Our findings predict that combining epigenetic drugs with traditional genotoxic therapies represents a rational strategy to target cancer cells, which frequently display HDR repair defects. In addition, activation of NHEJ, which is a prominent, error prone path in human cells, also contributes to the cytotoxic activity of HDACi in cancer cells. Future studies will be required to fully understand the contribution of NHEJ and HDR in directing tumor cell death by epigenetic agents; however, we have powerful genetic and cell based reporting tools to enhance our understanding of each repair pathway.

## Materials and Methods

### Chicken *RAD5* (human SHPRH homolog) Cloning and Gene Targeting Vectors

Two *RAD5* disruption constructs, RAD5-puro and RAD-bsr, were generated from genomic PCR products combined with puromycin and blasticidin selection marker cassettes. Genomic DNA sequences were amplified using SalI primer 5′-TATAAGTCGA- CAGGTTGGAATCTACCTTCTGGAAGCAGG-3′ and BamHI primer 5′-TATAAGGATCCTAGA- TTTTCACTCCATCCAGAGTAATGACC-3′ (for the left arm of the KO construct); and NotI primer 5′-TATAAGCGGCCGCTTTGCTACAACACTTATTTGTTGCAACAGG-3′ and BamHI primer 5′-TATAAGGATCCGAAATAGACGGCTTCTCAAAAGAACGCTGG3′ (for the right arm primer of the KO construct). Amplified PCR products were cloned into pBluescript II K. The BamHI site was used to clone marker gene cassettes. 0.4 kb of fragment from the genomic DNA amplified using the primers 5′-CAAAAAGATGACAGTCGTTCAAATCTAAATC- TGGG-3′ and 5′-CTTTAGAGCTTGACCCTCTCCAGG-3′ was used as a probe for Southern blot analysis.

### Human *FANCM* and *LIGIV* Targeting

The mutants were generated in the HCT116 cell background. The construction and phenotypes of *LIGIV*-null cells [43], [44] and *FANCM*-null cells [46] have been described.

### Cell Culture

Isogenic DT40 cell lines [30], each defective in a different DNA repair pathway (Table 1), were cultured in RPMI (Life Technologies) supplemented with 10% fetal bovine serum (FBS, Hyclone), 1% chicken serum (Life Technologies), 50 µM 2-mercaptoethanol (Sigma), 1 mM L-glutamine (Life Technologies) and 1% penicillin-streptomycin (Life Technologies). *BLM*- and *XPA*-defective lymphocytes (Coriell) were cultured in RPMI supplemented with 15% FBS and 2 mM L-glutamine. Wild-type human lymphocytes [49] were cultured in RPMI supplemented with 10% FBS, 1 mM L-glutamine, 1 mM sodium pyruvate (Life Technologies), 1% non-essential amino acids (Life Technologies), 50 µM 2-mercaptoethanol and 50 µg/ml gentamycin sulfate (Life Technologies). Wild-type and *SHPRH* mouse embryonic fibroblasts (MEFs) [50], RPE (ATCC), HEK293T (human embryonic kidney) (ATCC), HeLa (human cervical adenocarcinoma) (ATCC), HCT116 (human colorectal cancer cell) (ATCC), U2OS EJ2-GFP [40], isogenic HCT116 *FANCM*- [43], [46] and *LIG4*- [43], [44] null cells, HCC1937 cells complimented with a *BRCA1* cDNA and HCC1937 [45] were cultured in DMEM with Glutamax (Life Technologies) supplemented with 10% FBS and 1% penicillin- streptomycin. U2OS DR-GFP [34], [51] cells were cultured in McCoys modified 5A (Life Technologies) supplemented with 10% FBS, 2 mM L-glutamine and 1% penicillin-streptomycin. All cell lines were maintained in a humidified environment of 5% CO_2_ and 95% air at 37°C.

### Luciferase Assay

HEK293T ELG1-LUC cells [25] were plated at a density of 10,000 cells per well in a 96-well white, assay plate (Costar). 24 hr after seeding, cells were treated with 0.75 µM TSA or 50 µM SAHA and then incubated for an additional 48 hr. Either One-Glo luciferase reagent (Promega) to quantify luciferase activity or Cell-Titre Glo to quantify cell viability was added to each well. Luminescence intensity was measured using a Fluoroskan Ascent Luminometer (Thermo Scientific). Luciferase levels were normalized to cell viability.

### Protein Analysis

HEK293T cells were grown in 10-cm tissue-culture plates to ∼90% confluence and treated with 0.75 µM TSA, or DMSO (negative control) for 24 hr. To obtain total lysate, the cells were resuspended in lysis buffer [50 mM Tris, pH 7.5, 150 mM NaCl, 1% Nonidet P-40, 5 mM EDTA, protease inhibitors (Roche)] and lysed on ice for 30 min. Chromatin-bound fractions were isolated as described [52]. Proteins in the total lysate or the chromatin-bound fraction were separated by SDS-PAGE using a 4–15% tris-glycine gel (Bio-Rad) and transferred to a polyvinylidene difluoride membrane. Proteins in the membrane were detected by the ECL Western Blotting Detection System (GE Healthcare). HRP-conjugated anti-PCNA was purchased from Santa Cruz Biotechnology. Rabbit antiphospho-RPA32 (S4/S8), and rabbit antiphospho-CHK1 (S317) antibodies were purchased from Bethyl Laboratories. HRP-conjugated anti–β-tubulin antibody was purchased from Abcam. Rabbit antihistone H3 antibody was purchased from Upstate. Mouse antiphospho-histone H2AX (S140, 3F2) antibody was purchased from GeneTex.

### Pulsed-Field Gel Electrophoresis

HEK293T cells were treated with 100 uM TSA for 24 hours and then Pulsed-field gel electrophoresis was performed as described [25].

### siRNA Knockdown of *KU80* or *RAD51*

HeLa cells were transfected once with ON-TARGETplus NON-targeting siRNA or siRNA targeting *KU80* (Dharmacon) or twice with siRNA targeting *RAD51* (Dharmacon) using Lipofectamine RNAi Max (Life Technologies). 24 hr post-transfection, cells were treated with TSA (final concentration  =  0 µM to 0.04 µM, Sigma) or SAHA (final concentration  =  0 µM to 3.125 µM, Cayman Chemical Company). 48 hr following treatment, cell viability was determined using Cell Titer-Glo (Promega) according to the manufacturer's protocol. Viability was quantified on a Fluoroskan Ascent Luminometer (Thermo Scientific). Dose-response curves were generated using GraphPad Prism. Protein levels were visualized using antibodies against KU80 or RAD51 using whole cell extract prepared 72 hr after transfection.

### Viability Assays

All cells were cultured in 96-well white, assay plates (Costar). DT40 cells were plated at a density of 1,000 cells per well. TSA (final concentration  =  0 µM to 0.04 µM, Sigma) or SAHA (final concentration  =  0 µM to 3.125 µM, Cayman Chemical Company) were added immediately after plating and cells were treated for 48 hr. Human lymphoblasts were plated at a density of 20,000 cells per well. TSA (final concentration  =  0 µM to 1.5 µM) or SAHA (final concentration  =  0 µM to 100 µM) were added immediately after plating and cells were treated for 48 hr. MEFs and fibroblasts were plated at a density of 5,000 cells per well. TSA (final concentration  =  0 µM to 1.5 µM) or SAHA (final concentration  =  0 µM to 100 µM) were added 24 hr after plating and cells were treated for 48 hr. Following treatment, cell viability was determined using Cell Titer-Glo (Promega) according to the manufacturer's protocol. Viability was quantified on a Fluoroskan Ascent Luminometer (Thermo Scientific). Dose-response curves were generated using GraphPad Prism.

### Sister Chromatid Exchange (SCE) Assay

SCE was carried out as described [25]. Briefly, RPE cells were treated with 0.2 µM TSA for 48 hr. Cells were washed with PBS and then cultured in the presence 5 µg/ml BrdU for an additional 48 hours. Colcemid (0.05 µg) was added 3 hr prior to preparation of metaphase spreads. Metaphase spreads on slides were prepared by standard hypotonic treatment and fixation with methanol and glacial acetic acid and incubated at 45°C for 18 hr before staining with 2 mg/ml Hoechst 33258 in 2x SSC solution. The slides, mounted with Sorensen's phosphate buffer pH 7.0, were then treated by UV light for 30 min and subsequently incubated at 65°C for 1 hr in 2x SSC. Chromosomes on slides were stained with 4% Giemsa in Sorenson's buffer pH 6.8 for 30 min and monitored under microscope. Images of 50 metaphase spreads for each cell line were captured from 2 independent experiments.

### Immunofluorescence and Microscopy

5×10^4^ RPE cells were plated in each well of a 4-well chamber slide (Nunc). 24 hr after plating, cells were treated for an additional 24 hr with 2 mM HU or 1 hr with either 0.5 µM TSA or 32 µM SAHA. Following treatment, slides were washed with PBS then fixed with 4% paraformaldehyde/0.2% Triton X-100 in PBS for 20 min. After a brief wash with PBS, slides were permeabilized with 0.5% NP40 in PBS for 10 min and then washed with 0.5% BSA/0.175% Tween-20 in PBS. Slides were blocked for 1 hr in 1% donkey serum/2% BSA in PBS and then incubated in rabbit antiphospho-53BP1-Ser1778 (Cell Signaling) diluted 1∶1000 in 3% BSA in PBS for 2 hr. After extensive washes with 0.5% BSA/0.175% Tween-20 in PBS, slides were incubated for 1 hr in Alexa-Fluor 488-conjugated goat anti-rabbit IgG secondary antibody (Invitrogen) diluted 1∶250 in 3% BSA in PBS. Following washes in 0.5% BSA/0.175% Tween-20 in PBS, slides were mounted with ProLong Gold Antifade Reagent with DAPI (Life Technologies). Wide-field images were collected using a Personal DeltaVision system (Applied Precision Inc., Issaquah, WA, USA) mounted on an inverted Olympus IX71 microscope with an oil immersion UPlanSApo 100×/1.40 objective lens. All images were acquired using a CoolSNAP ES2 camera with 1×1 binning. Excitation filters 390/18 and 490/20 with corresponding emission filters 435/48 and 528/38 were used to collect the DAPI and Alexa-Fluor 488. All images were collected using the same settings and all were deconvolved using an iterative constrained method with 10 cycles, medium noise filtering in SoftWoRx version 4.0.0. At least 145 cells were imaged from each treatment.

### Recombination Reporter Assays

A U2OS cell line stably transfected with a single copy of an intact DR-GFP reporter gene (gift from Dr. M. Jasin at the Memorial Sloan-Kettering Institute) was used to measure the HDR frequency [34], [51]. A single DSB was introduced by expressing the I-SceI endonuclease. Cells were treated with 0.25 µM TSA at 24 hr after transfection. After a 48 hr treatment, cells were analyzed on a BDFACSCalibur with a 15 mW 488 nm argon laser. GFP emission was detected with a 530/30 band-pass filter and DsRed was detected with a 670LP filter. Data was analyzed using FlowJo. The HDR frequency was determined by the number of cells expressing GFP divided by the number of cells expressing DsRed (as an indicator for transfection). Experiments were repeated at least 3 times, and the average values are reported.

The Hela cell line iHO4.5GFP was used to measure the HDR frequency. This cell line contains the DR-GFP reporter twin cassettes (GFP-I, GFP-II) that are mutated and GFP minus. Adjacent to the Cassette I, a unique I-SceI site was engineered and the I-SceI gene introduced and placed under control of the Tet-on promoter. Cells were either continuously exposed to doxycycline (Dox) for 7 days, (new Dox added each day with medium change) or pulsed with Dox for 24 hr and then washed and chased in Dox-free medium for 7 days. HDR frequency was measured by FACS analysis and reported as the percentage of GFP cells. The percent GFP readout reveals the HDR frequency, based on Q-PCR analyses, as shown previously [23].

### Non-Homologous End Joining Reporter Assays

Two different clones of NHEJ reporter cells were constructed and tested (IHN20.22 and IHN20.41 with similar results using other clones, not shown). The NHEJ based reporter is based on the construct from Mao et al. [39] and consists of a single GFP cassette disrupted by an internal adenoviral exon and a Neomycin selectable marker. The cassette also includes the CMV promoter; however, due to the exon insertion, the cells are GFP minus. Flanking the adenoviral exon are twin I-Sce1 sites that were placed in an inverted orientation. Digestion with the homing endonuclease I-Sce1, creates a DS DNA break with incompatible ends, which are repaired by NHEJ. To create an inducible I-SceI system, a stably transfected HeLa cell lines were made containing a single integrated copy of the NHEJ (GFP) cassette. The construct was validated by sequencing and qPCR to confirm copy number. Two lentiviruses (pLV-TetO-HA-Sce1 and rtTA) were used to co-infect this stable line. A number of clones were screened to identify a single copy GFP cassette line that was induced by doxycycline addition in a Tet-on format and two clones were selected for this study. Following induction of I-SceI by addition of doxycycline, DS DNA breaks are produced that flank the adenoviral exon. The breaks can only be repaired by the NHEJ pathway (not HDR) and in some repair products WT GFP is produced [53], [54]. The percent GFP was determined by FACS analysis as previously described [22], [23]. For this analysis, 2×10^5^ IHN20.22 or IHN20.41 cells were plated in 35 mm dishes, plus or minus Dox (or DMSO negative controls). Cells were pulsed with 0.5 ug Doxycycline/mL for 24 hours to induce a detectable and synchronous wave of NHEJ repair. At this time, TSA at the indicated concentrations was added and the GFP measured as a time course over 3 days by FACS analysis. Induction of I-SceI gene expression was not affected by HDACi treatment, using this approach. NHEJ was also analyzed using a complementary approach with a U2OS cell line carrying an End Joining (EJ-5) reporter in genome [40]. To measure NHEJ, 1×10^5^ U2OS EJ2-GFP cells were plated in each well of a 6-well plate, and transfected the next day with 3 µg of I-SceI expression vector (pCGA-I-SCE) and 10 µl of Lipofectamine LTX (Life Technologies) in culture medium without antibiotics. 24 hr after transfection, cells were treated with either DMSO as a negative control, or with 100 nM TSA. NHEJ frequencies were measured by FACS analysis (48 hr post-treatment) to reveal the total percent GFP. siRNA knockdown was performed one time 24 hours prior to transfection of I-SceI with ON-TARGETplus NON-targeting siRNA, siRNA targeting *KU80* (Dharmacon) or siRNA targeting *53BP1* (Dharmacon) using Lipofectamine RNAi Max (Life Technologies). Protein levels were visualized using antibodies against KU80 or 53BP1 using whole cell extract prepared 72 hr after transfection.

### Statistical Analyses

The Graphpad Prism 5 (Graphpad Prism®) software package was used to perform statistical analyses.

### Densitometry Analyses

AlphaView SA (Cell Biosciences) software was used to perform densitometry analyses.

## Supporting Information

Figure S1 (A) HeLa iHO4.5GFP cells were pulsed for 24 hr with Dox, washed and then chased in the presence of the indicated amounts of SAHA. The graph represents the average percent GFP ± SD. The difference between +Dox/−SAHA and +Dox/+SAHA not significant and was determined using a two-tailed t-test. (B) *BLM* and *XPA* human lymphocytes were treated with 0 to 118 mM MMS for 48 hr. Viability was determined using CellTiter-Glo. (C) *FANCM* human colorectal cancer cells were treated with 0 to 118 mM MMS for 48 hr. Viability was determined using CellTiter-Glo. (D) 72 hr after transfection with either non-targeting siRNA or siRNA against KU86, protein levels were visualized using antibodies against KU86. Relative densitometry is shown on right. (E) 72 hr after transfection with either non-targeting siRNA or siRNA against RAD51, protein levels were visualized using antibodies against RAD51. Relative densitometry is shown on right.
(TIF)

Figure S2 (A) HeLa IHN20.41 cells were pulsed for 24 hr with Dox, washed and chased in the presence of the indicated amounts of SAHA for 3 days. The percentage of GFP-positive cells was determined by FACS analysis. The graph represents the average percent GFP ± SD. Significance between +Dox/−SAHA and +Dox/+SAHA was calculated using a two-tailed t-test. (B) U2OS EJ2-GFP cells were transfected with pCGA-I-SCEI and then treated with 100 nM TSA for 48 hr. The graph represents the average NHEJ frequency ± SD following treatment determined by FACS analysis. The difference between non-treated and TSA treated was not significant and was determined using a one-tailed t-test. (C) U2OS EJ2-GFP cells were transfected with non-targeting siRNA or siRNA targeting *KU80* or *53BP1*. 24 hr after knockdown, the cells were transfected with pCGA-I-SCEI and then treated 24 hr later with 100 nM TSA for 48 hr. The graph represents the average NHEJ frequency ± SD following treatment determined by FACS analysis. The difference between non-targeting and *KU80* knockdown was significant and was determined using a two-tailed t-test. The difference between non-targeting and *53BP1* knockdown was not significant and was determined using a two-tailed t-test. (D) 72 hr after transfection with either non-targeting siRNA, siRNA against Ku80 or siRNA against 53BP1, protein levels were visualized using antibodies against indicated proteins. Relative densitometry is shown on right. (E) Analysis of SAHA induced NHEJ in iHN20.22 cells. The NHEJ reporter cells were not treated with Doxycycline in order to assess direct genotoxicity and NHEJ recovery in the absence of directed DSBs at the I-Sce1 site. Cells were incubated for 24 hr. in the indicted concentrations of SAHA and percent GFP was determined by FACS ± SD.
(TIF)
