# Supplementary figures and images for: Histone Deacetylase Inhibitors Selectively Target Homology Dependent DNA Repair Defective Cells and Elevate Non-Homologous Endjoining Activity
Source: PLoS One. 2014 Jan 23;9(1):e87203. doi: 10.1371/journal.pone.0087203 (PMC3900704; doi:10.1371/journal.pone.0087203)

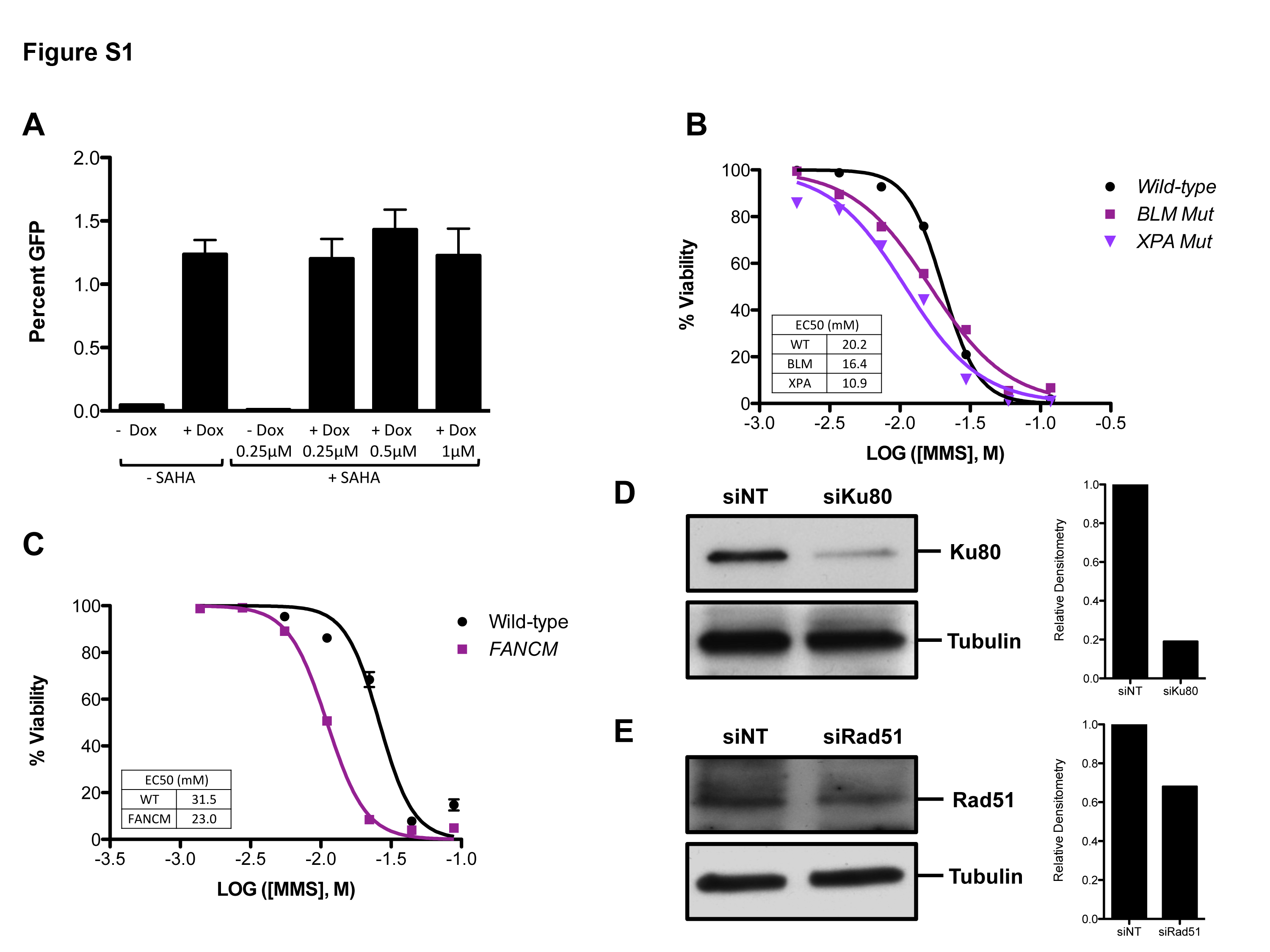

Supplement: Figure S1 — (A) HeLa iHO4.5GFP cells were pulsed for 24 hr with Dox, washed and then chased in the presence of the indicated amounts of SAHA. The graph represents the average percent GFP ± SD. The difference between +Dox/−SAHA and +Dox/+SAHA not significant and was determined using a two-tailed t-test. (B) BLM and XPA human lymphocytes were treated with 0 to 118 mM MMS for 48 hr. Viability was determined using CellTiter-Glo. (C) FANCM human colorectal cancer cells were treated with 0 to 118 mM MMS for 48 hr. Viability was determined using CellTiter-Glo. (D) 72 hr after transfection with either non-targeting siRNA or siRNA against KU86, protein levels were visualized using antibodies against KU86. Relative densitometry is shown on right. (E) 72 hr after transfection with either non-targeting siRNA or siRNA against RAD51, protein levels were visualized using antibodies against RAD51. Relative densitometry is shown on right. (TIF) [file pone.0087203.s001.tif]

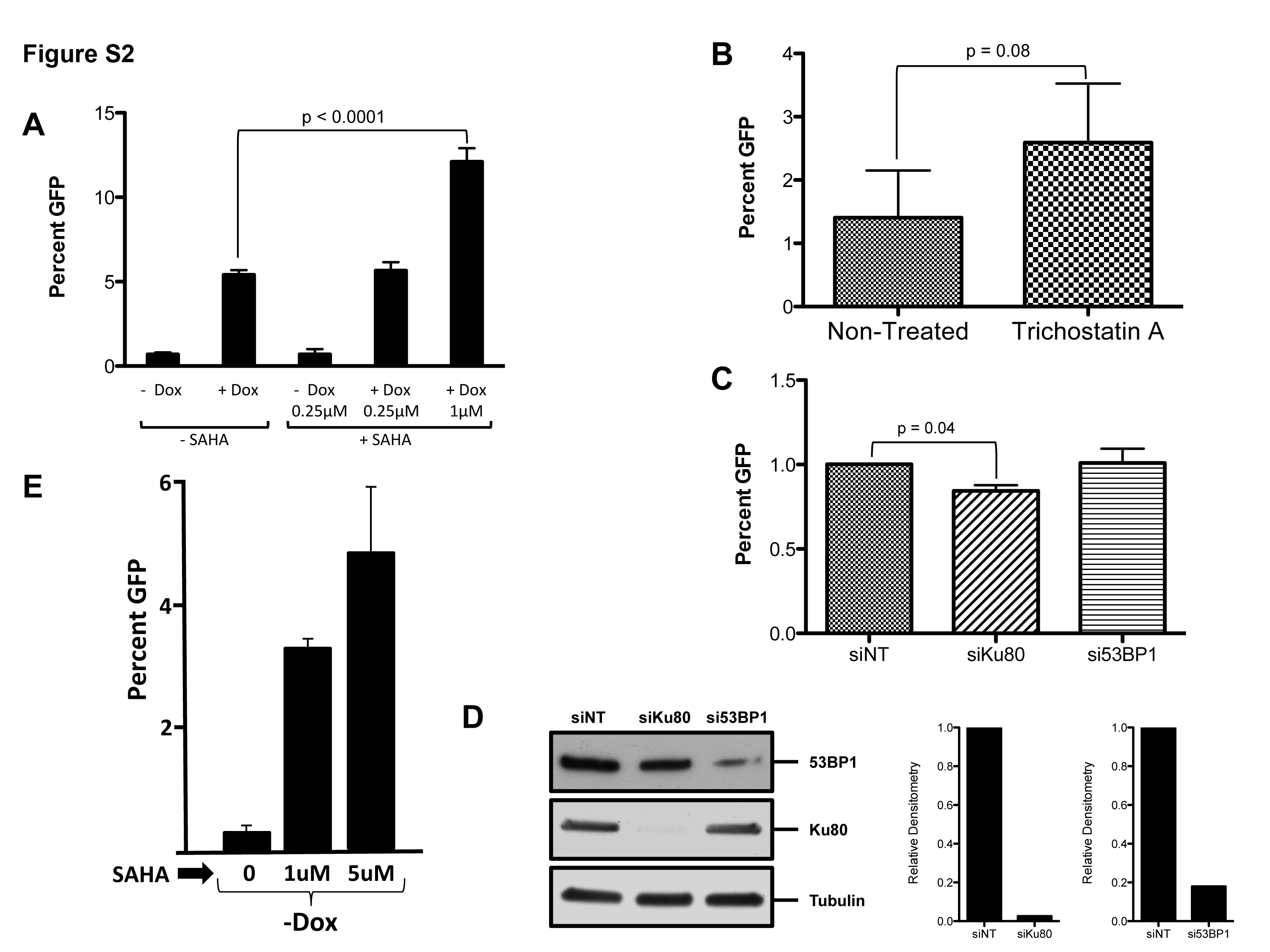

Supplement: Figure S2 — (A) HeLa IHN20.41 cells were pulsed for 24 hr with Dox, washed and chased in the presence of the indicated amounts of SAHA for 3 days. The percentage of GFP-positive cells was determined by FACS analysis. The graph represents the average percent GFP ± SD. Significance between +Dox/−SAHA and +Dox/+SAHA was calculated using a two-tailed t-test. (B) U2OS EJ2-GFP cells were transfected with pCGA-I-SCEI and then treated with 100 nM TSA for 48 hr. The graph represents the average NHEJ frequency ± SD following treatment determined by FACS analysis. The difference between non-treated and TSA treated was not significant and was determined using a one-tailed t-test. (C) U2OS EJ2-GFP cells were transfected with non-targeting siRNA or siRNA targeting KU80 or 53BP1. 24 hr after knockdown, the cells were transfected with pCGA-I-SCEI and then treated 24 hr later with 100 nM TSA for 48 hr. The graph represents the average NHEJ frequency ± SD following treatment determined by FACS analysis. The difference between non-targeting and KU80 knockdown was significant and was determined using a two-tailed t-test. The difference between non-targeting and 53BP1 knockdown was not significant and was determined using a two-tailed t-test. (D) 72 hr after transfection with either non-targeting siRNA, siRNA against Ku80 or siRNA against 53BP1, protein levels were visualized using antibodies against indicated proteins. Relative densitometry is shown on right. (E) Analysis of SAHA induced NHEJ in iHN20.22 cells. The NHEJ reporter cells were not treated with Doxycycline in order to assess direct genotoxicity and NHEJ recovery in the absence of directed DSBs at the I-Sce1 site. Cells were incubated for 24 hr. in the indicted concentrations of SAHA and percent GFP was determined by FACS ± SD. (TIF) [file pone.0087203.s002.tif]
